# The nasal microbiome mirrors and potentially shapes olfactory function

**DOI:** 10.1038/s41598-018-19438-3

**Published:** 2018-01-22

**Authors:** Kaisa Koskinen, Johanna L. Reichert, Stefan Hoier, Jochen Schachenreiter, Stefanie Duller, Christine Moissl-Eichinger, Veronika Schöpf

**Affiliations:** 1 Department of Internal Medicine, Medical University of Graz, Graz, Austria; 2 BioTechMed-Graz, Graz, Austria; 3 Institute of Psychology, University of Graz, Graz, Austria; 4 Hanusch Krankenhaus Vienna, Department of Otorhinolaryngology, Head and Neck Surgery, Vienna, Austria; 5 ENT-practice Graz, Graz, Austria

## Abstract

Olfactory function is a key sense for human well-being and health, with olfactory dysfunction having been linked to serious diseases. As the microbiome is involved in normal olfactory epithelium development, we explored the relationship between olfactory function (odor threshold, discrimination, identification) and nasal microbiome in 67 healthy volunteers. Twenty-eight subjects were found to have normal olfactory function, 29 had a particularly good sense of smell (“good normosmics”) and 10 were hyposmic. Microbial community composition differed significantly between the three olfactory groups. In particular, butyric acid-producing microorganisms were found to be associated with impaired olfactory function. We describe the first insights of the potential interplay between the olfactory epithelium microbial community and olfactory function, and suggest that the microbiome composition is able to mirror and potentially shape olfactory function by producing strong odor compounds.

## Introduction

The human olfactory system is able to discriminate a vast number of odors^1^. This function is mediated by olfactory receptors situated within the olfactory epithelium. The sense of smell shapes our perception of our external environment and is essential in decision-making and in guiding behavior, including eating behavior, and detection of danger, as well as contributing significantly to the hedonic component of everyday life (e.g. flavor and fragrance perception^2,3^).

Anosmia (complete loss of olfactory function) and hyposmia (decreased olfactory function) together affect approximately 20% of the population^3^. The risk of olfactory dysfunction increases with age, and the condition may also result from chronic sinonasal diseases, head trauma, upper respiratory infection or neurodegenerative diseases.

Negative effects on mood, enjoyment of food, safety, personal hygiene, social interactions, and sexual life have been reported in individuals with olfactory impairment^4–7^ (for review see^8^). Even mild olfactory impairment can negatively influence interpersonal relationships as shown for patients with traumatic brain injury^9^. The effect of olfactory dysfunction on bodyweight is unclear, with some studies reporting weight gain^10–12^ and others weight loss^13,14^. A study in 176 individuals with olfactory dysfunction found that 21% reported weight gain, 11% reported weight loss, and the rest reported no change^15^. Weight gain was more common in those with a better ability to identify odors and in younger subjects^15^.

Importantly, olfactory deficits have also been identified as indicators of several neurologic diseases, including multiple sclerosis, epilepsy, and possibly, Alzheimer’s and Parkinson’s disease^16,17^. They also represent ultra-high risk indicators for psychosis^18^. Very recently, olfactory dysfunction has been identified as the best predictor for 5-year mortality in older adults^19^; outperforming such factors as heart failure, stroke, diabetes, hypertension, liver damage and even cancer^20^.

The human microbiota, consisting of 3.8 × 10^13^ microorganisms^21^ mainly associated with the colon (3.8 × 10^13 ^^22^) and skin (10^11 ^^23^), fulfils numerous important tasks, ranging from food degradation to playing a key role in host immunity^24^. These microorganisms are known to interact with the body mostly via their secondary metabolites, which include hormone-like molecules^25^, short-chain fatty acids - the end products of gut bacterial fermentation - and serotonin precursors^26^. Dysbiosis (imbalance of the human microbial community), has been associated with a number of diseases, including cancer, metabolic disorders, inflammatory bowel disease, depression and many others. Thus, the microbial community has the capability to reflect health status and functionality, and may have potential as a diagnostic tool^27,28^.

While potentially important with respect to disorders such as chronic rhinosinusitis^29^, the nasal microbial community remains largely understudied. However, a recent report indicates that microorganisms contribute to normal development of the olfactory epithelium, as germ-free mice had a thinner olfactory cilia layer and reduced levels of olfactory epithelium cell apoptosis and proliferation^30^. In a recent study, focusing on the oral and nasal microbiome of Parkinson’s disease patients and a control group, no notable differences in nasal bacterial abundance or diversity were found, but overall olfactory function was associated with the abundance of certain bacterial taxa, including *Moraxella* and *Staphylococcus*^31^.

Based on these preliminary insights, we became interested in the relationship of nasal microbiome and olfactory function. We propose that the bacterial composition of the nasal olfactory epithelium *per se* can mirror olfactory function. To this end, the present study was implemented to explore correlations between the nasal microbial community composition and olfactory performance measures in healthy individuals. Briefly, we assessed olfactory function (odor threshold, discrimination, identification) in 67 healthy volunteers using the Sniffin’ Sticks test battery (Burghart Instruments, Wedel, Germany) and explored the relationship between their olfactory performance and nasal microbiome. As additional parameters, we as well included sex and BMI as potential factors for olfactory function and microbiome composition.

## Results

### Study cohort parameters

**Figure 1:**
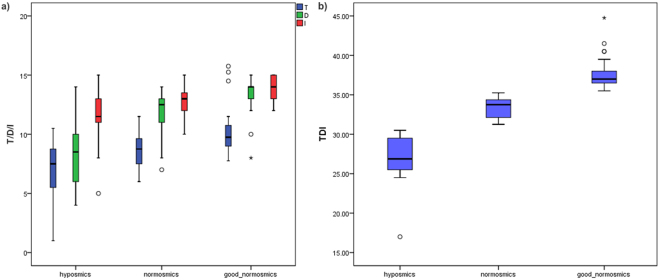
Sense of smell of the study cohort. (**a**) Boxplots showing odor threshold (T), discrimination (D) and identification (I) scores for each of the three groups (good normosmics, hyposmics and normosmics based on total TDI scores). (**b**) Boxplots showing the distribution of total TDI scores in each of the three groups (good normosmics, hyposmics and normosmics based on total TDI). Outliers are marked with circles, far outliers with stars.

Overall, our cohort comprised 50 women and 17 men (mean weight 66.7 kg [SD = 13.4], and mean BMI 22.7 [SD = 3.8]): 53 were normal weight (BMI 18.5–25), 10 overweight (BMI > 25), and 4 underweight (BMI < 18.5). As shown in Supplementary Table 1, 57 were found to be normosmic and 10 hyposmic. Based on total (odor threshold: T, discrimination: D, identification: I) TDI scores, 29 of the normosmic subjects were categorized as “good normosmics” (see Fig. 1 for the olfactory scores and their distribution for each subgroup). We also classified subjects according to subscores for T, D, and I; see Supplementary Table 1). Across the whole dataset, there was no correlation (Spearman’s rho) between olfactory scores and age or BMI (Table 1, see Supplementary Figures 1 and 2 for distribution of BMI and age for each subgroup). In average normosmics, there was a marginal negative correlation between total TDI scores and age (r_s_ = −0.37, p = 0.052). In good normosmics, there was a nonsignificant correlation between olfactory discrimination scores and age (r_s_ = 0.33, p = 0.08). The only significant sex difference we observed was a marginal difference in BMI (t(65) = 1.7, p = 0.096), between men (mean 24.0, SD = 4.2) and women (mean 22.3, SD = 3.6). Olfactory scores did not differ significantly between men and women.

**Table 1:** Correlations between olfactory performance scores, age, and BMI.

	TDI	T	D	I	Age	BMI
TDI	1.000	0.546^**^	0.675^**^	0.603^**^	0.022	−0.153
	—	<0.001	<0.001	<0.001	(0.857)	(0.215)
Threshold (T)	0.546^**^	1.000	0.001	0.149	0.031	−0.206
	<0.001	—	(0.993)	(0.229)	(0.802)	(0.095)
Discrimination (D)	0.675^**^	0.001	1.000	0.192	0.075	0.066
	<0.001	(0.993)	—	(0.120)	(0.546)	(0.593)
Identification (I)	0.603^**^	0.149	0.192	1.000	0.073	−0.133
	<0.001	(0.229)	(0.120)	—	(0.558)	(0.283)
Age	0.022	0.031	0.075	0.073	1.000	0.359^**^
	(0.857)	(0.802)	(0.546)	(0.558)	—	(0.003)
BMI	−0.153	−0.206	0.066	−0.133	0.359^**^	1.000
	(0.215)	(0.095)	(0.593)	(0.283)	(0.003)	—

Correlation coefficients (Spearman’s rho) are shown, with p-value in brackets.

**Statistically significant correlation; p ≤ 0.01 (2-tailed).

### Typical nasal microbiome was found

**Figure 2:**
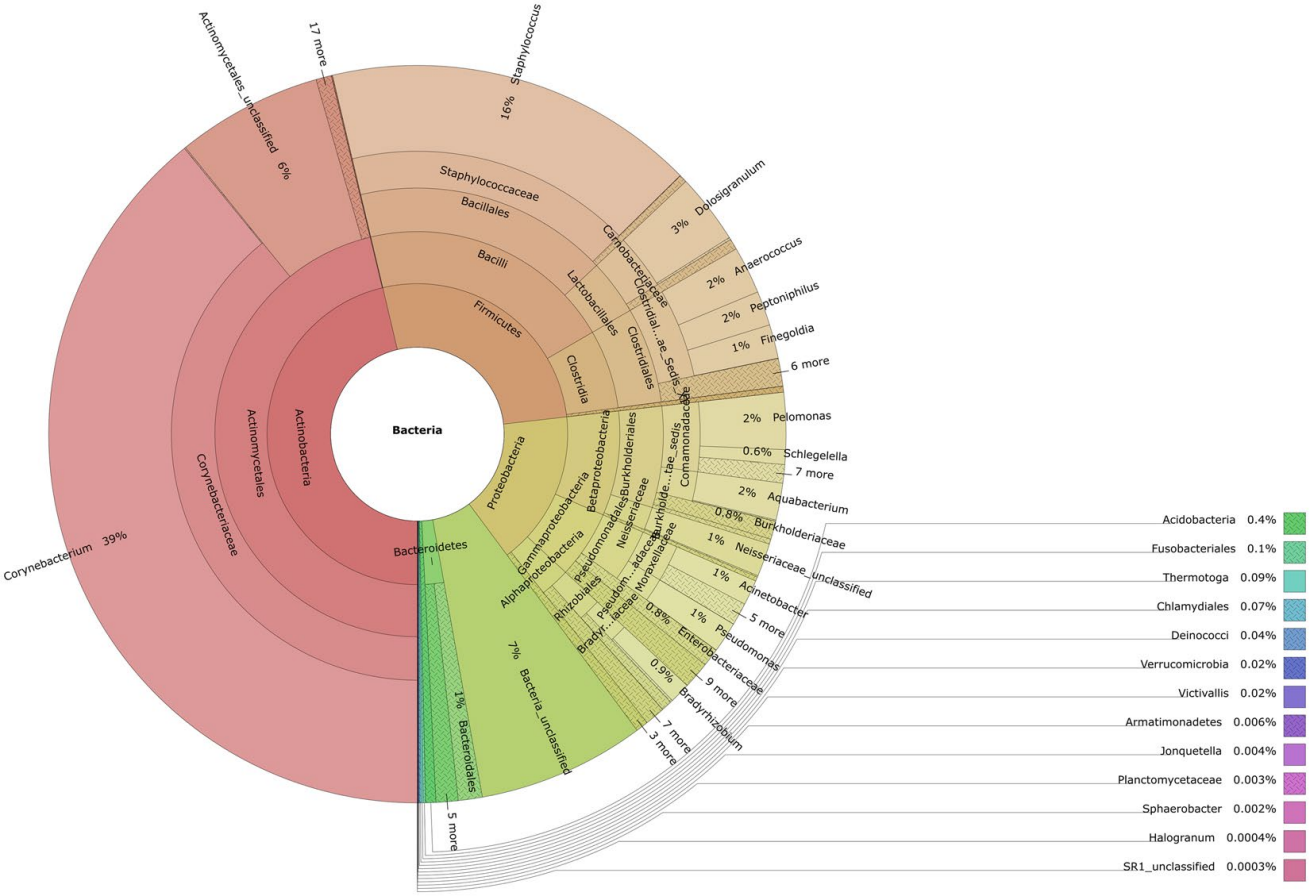
Hierarchical display of the human nasal bacteriome. Krona diagram visualization shows that the nasal microbiome of study subjects was dominated by typical skin and respiratory tract associated bacteria such as *Corynebacterium*, *Staphylococcus* and *Dolosigranulum* (normalized data from all study participants).

We characterized the nasal microbiome of the study subjects using Illumina MiSeq next generation sequencing (Supplementary Table 2). We identified 27 phyla, four belonging to the phylum *Archaea* (*Thaumarchaeota* [*Nitrososphaera]*, *Euryarchaeota* [*Methanosphaera*, *Methanobrevibacter*, and *Halogranum], Pacearchaeota* and unclassified *Archaea*) and 23 to *Bacteria*. The most abundant phyla were *Actinobacteria* (50% of all sequence reads), *Firmicutes* (28%), and *Proteobacteria* (14%). The fourth most abundant bacterial phylum, *Bacteroidetes*, was represented by only 1.5% of all sequence reads. The most abundant bacteria at genus level were *Corynebacterium* (43%), a typical human skin bacterium^32,33^ also frequently found in the nose^34,35^, *Staphylococcus* spp. (15%), characteristic nasal residents^34,36^, and *Dolosigranulum* (4%), commensal inhabitants of the upper respiratory tract that have been implicated in causing infections^37^ but have also been identified as health-associated commensals^38^. Additionally we detected *Peptoniphilus* (4%), which is known to be part of the vaginal and gut microbiota^39,40^ and is among the most abundant bacteria in nasal samples, based on 16 S rRNA amplicon sequencing^41^, and culture^42^. Hyposmics had a higher proportion of *Peptoniphilus* (10%); this, however, was mainly driven by one subject (no. 49), who showed a predominant presence of signatures from this genus (see Supplementary Figures 3, 4 and 5, and Supplementary Table 2). An overview of the 300 most commonly detected bacterial genera that we identified is given in Fig. 2.

**Figure 3:**
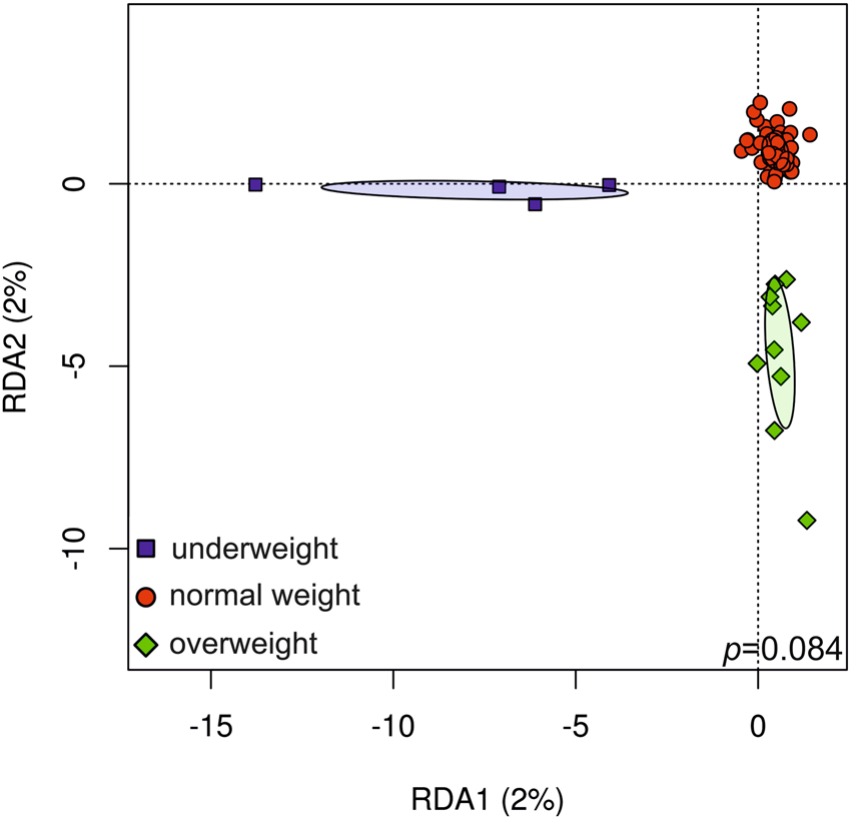
Sense of smell and body mass index. RDA plot depicting the grouping of nasal microbial communities of normal weight (BMI 18.5–25), overweight (>25), and underweight (<18.5) study subjects at OTU level (p = 0.084).

As BMI has previously been linked to olfactory function, we expected that subjects with differing BMI might also carry dissimilar nasal microbial communities. Indeed, the nasal microbiome did differ between those with normal, high and low BMI (Fig. 3), as the microbiome samples grouped separately. However, the differences were not found to be statistically significant (probably because of the small sample size), but a trend was obvious from a p-value of 0.084. The small sample size particularly for the underweight study subjects could potentially also explain why the association between BMI and olfactory function was not detected in this study.

### Microbial community composition differs between normosmics and hyposmics

**Figure 4:**
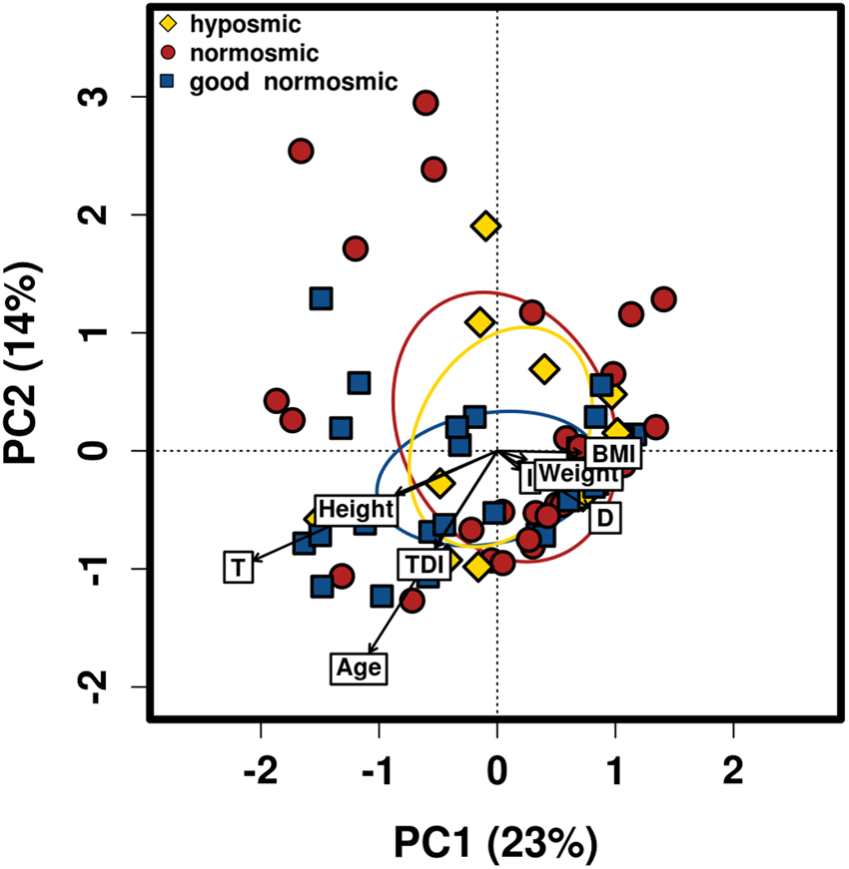
Nasal microbiome and olfactory function. PCA ordination presents an overview of relationship between nasal microbiome in hyposmic, normosmic and good normosmic study subjects and the collected metadata indicated by arrows.

We compared the microbial communities between normosmics and hyposmics using alpha and beta diversity measures to assess the relationship between olfactory function and nasal microbiome. An overview of relationship between nasal microbiome in hyposmic, normosmic and good normosmic study subjects and the collected metadata is presented in Fig. 4.

**Figure 5:**
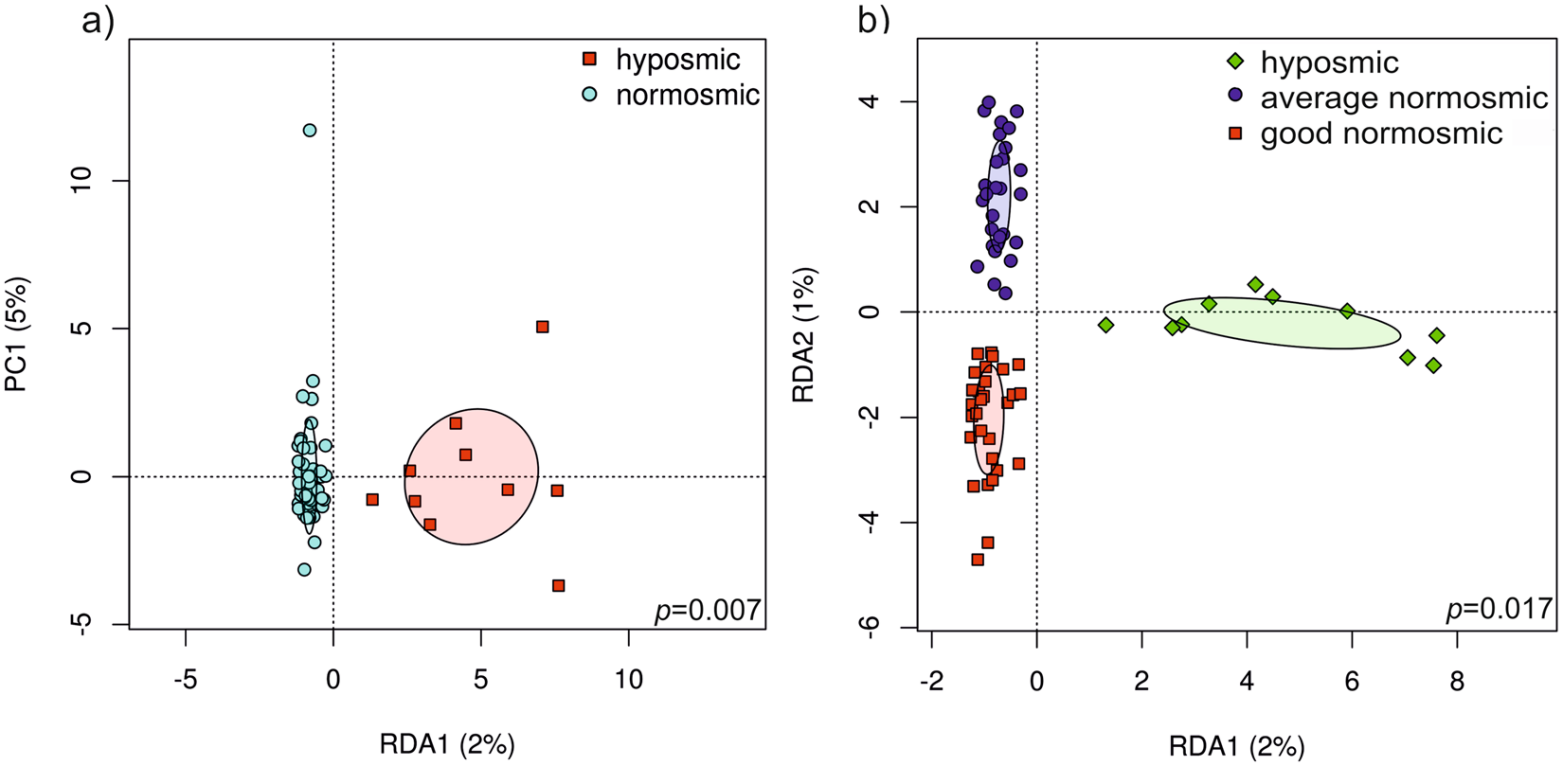
Sense of smell and nasal microbial community structure are interrelated. (**a**) RDA plot depicting the nasal microbial community grouping of (**a**) normosmic and hyposmic study subjects, and (**b**) average normosmics, hyposmics and good normosmics at OTU level.

Normosmic and hyposmic subjects were found to have significantly different nasal microbial community composition in the redundancy analysis (RDA) plot, both at operational taxonomic unit (OTU) (p = 0.007) (Fig. 5a) and genus (p = 0.037) levels. Furthermore, when the normosmic group was divided into average and good normosmics, there were significant differences between the 3 groups (hyposmics, average normosmics and good normosmics) at OTU level (p = 0.017) and near-significant differences at genus level (Fig. 5b). However, diversity (richness and evenness) of the nasal microbiome was not associated with overall olfactory function (based on total TDI score).

### Microbial community composition and diversity reflects odor discrimination and threshold

We also evaluated whether subcategories of total olfactory function, i.e odor identification, threshold, and discrimination, are particularly associated with the dissimilarities that we detected in nasal microbial community structure.

**Figure 6:**
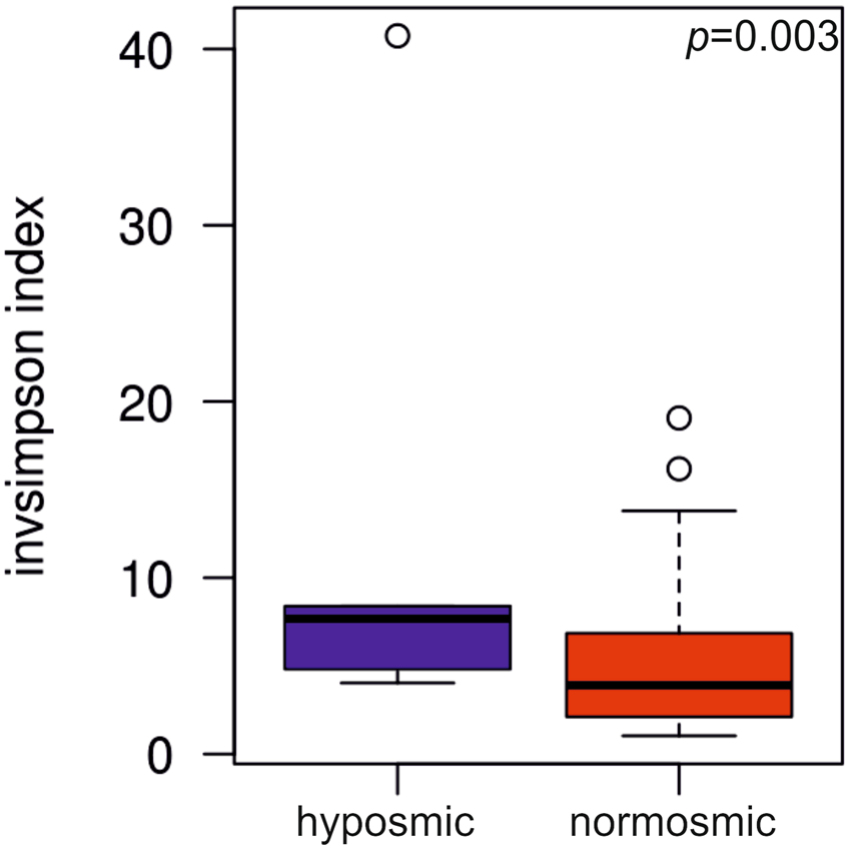
Odor threshold is associated with nasal microbiome diversity. Boxplot depicting the nasal microbial diversity of study subjects with good normosmic, normosmic and hyposmic odor thresholds at OTU level (ANOVA, p = 0.01).

The results show that odor identification was not associated with nasal microbial community structure or diversity, but some associations were found for odor threshold and discrimination. Subjects with hyposmia regarding odor threshold had higher microbial diversity (inverse simpson index) compared to normosmics (p = 0.003) (Fig. 6). Additionally, when the normosmic group was further divided into average and good normosmics (for threshold) the groups differed significantly (p = 0.01) in diversity. However, no significant difference was detected in diversity between average and good normosmics regarding odor threshold (p = 0.493).

**Figure 7:**
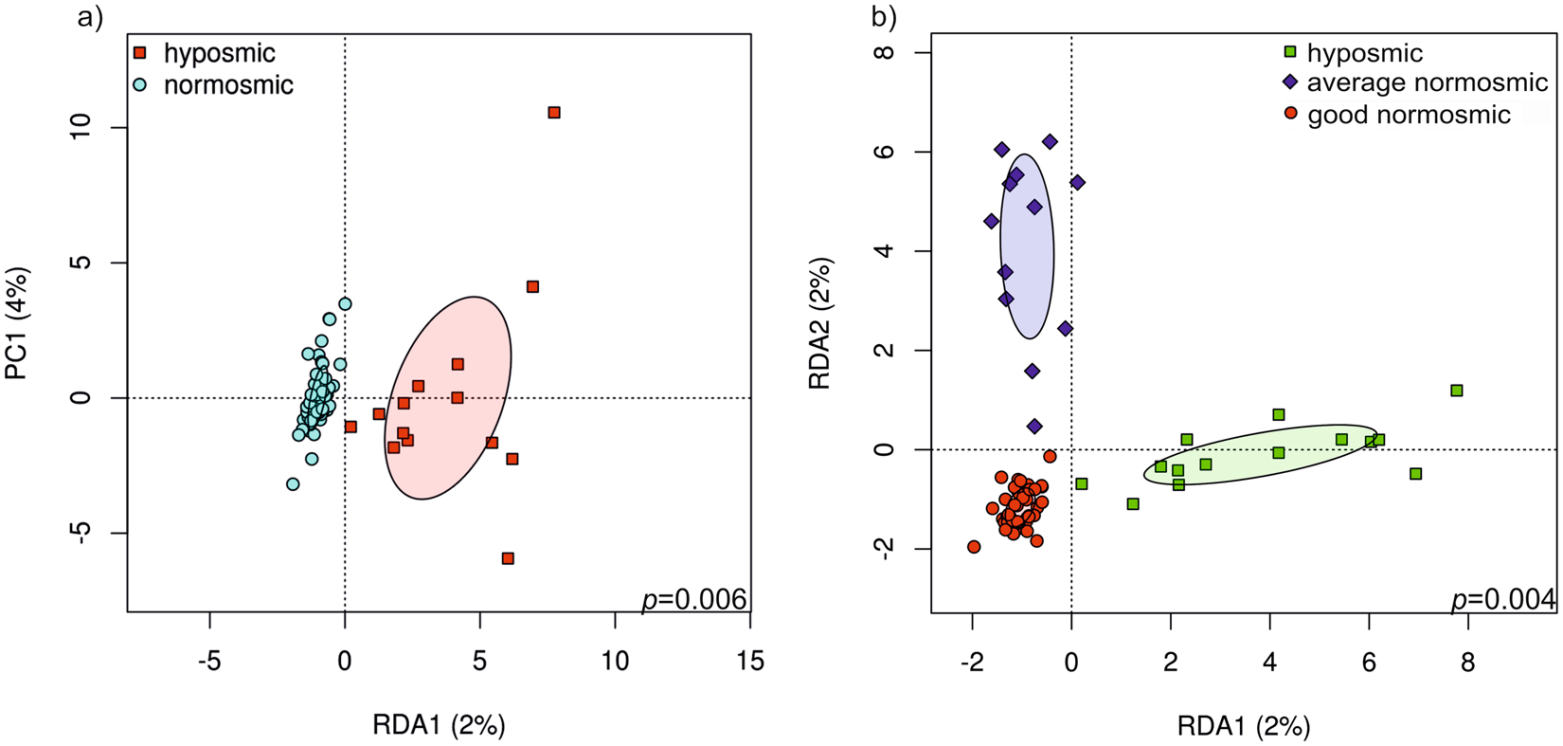
Odor discrimination is associated with nasal microbial community structure. RDA plot depicting the grouping of nasal microbial communities of (**a**) normosmics and hyposmics, and (**b**) good normosmics, average normosmics, and hyposmics regarding odor discrimination at OTU level.

Odor discrimination was related to nasal microbiome community composition (Fig. 7), with significant differences between hyposmic versus normosmic subjects (p = 0.006) (Fig. 6a), as well as between hyposmics, average and good normosmics (p = 0.004) (Fig. 7b), and nearly significant difference also between the average versus good normosmics (p = 0.075).

### Decreased olfaction is associated with specific bacterial taxa

To investigate individual OTUs were correlated with total TDI scores in the dataset, we applied regression analysis (Pearson correlation), but no significant result was obtained. When we looked specifically at odor threshold score, the relative abundance of one OTU belonging to genus *Campylobacter* correlated negatively and significantly with odor threshold (Pearson correlation, p = 0.029). However, the relative abundance of any described OTU in this study did not correlate significantly with odor discrimination and identification scores. As the odor threshold, identification and discrimination seem to associate differently to the nasal microbiome (Fig. 4), we analysed the connections separately (Table 2, Supplementary Table 3) using LEfSe (Linear discriminant analysis Effect Size^43^) algorithm. LEfSe allows identification of features (e.g. taxons or functions) that most likely explain differences between groups. Our dataset was subjected to LEfSe analysis for determination of specific microbial markers for the subcategories odor threshold, identification and discrimination. Overall, for all subcategories, a number of specific microbial signatures were identified. I.e. low odor identification capability was associated with signatures of Proteobacteria, Actinobacteria, Firmicutes, and Bacteroidetes, as shown in Table 2 and Supplementary Table 3, which summarize the microbial genera significantly associated with olfactory function subcategories.

**Table 2:** Microbial genera associated with olfactory function subcategories.

Phylum	Class	Family	Genus	hyposmic discrim-mination	normosmic discrim-mination	hyposmic threshold	normosmic threshold	hyposmic identification	normosmic identification
*Actinobacteria*	*Actinobacteria*	*Actinomycetaceae*	*Actinomyces*			**♦♦**		♦	
		*Actinomycetales_*uncl	*Actinomycetales_*uncl			♦		♦	
		*Cellulomonadaceae*	*Cellulomonas*			♦		♦	
		*Corynebacteriaceae*	*Corynebacterium*	**♦♦** **♦**	♦	**♦♦♦♦♦**			
		*Microbacteriaceae*	*Microbacteriaceae_*uncl			♦		♦	
		*Propionibacteriaceae*	*Propionibacterium*			♦		♦	
*Bacteria_*uncl	*Bacteria_*uncl	*Bacteria_*uncl	*Bacteria_*uncl	**♦♦**		♦			
*Bacteroidetes*	*Bacteroidia*	*Rikenellaceae*	*Alistipes*			♦		♦	
		*Bacteroidales_*uncl	*Bacteroidales_*uncl			**♦♦**		♦	
		*Bacteroidaceae*	*Bacteroides*	**♦♦**		**♦♦**			
		*Porphyromonadaceae*	*Porphyromonas*	♦		♦		♦	
		*Prevotellaceae*	*Prevotella*	♦	♦	♦			
	*Flavobacteriia*	*Flavobacteriaceae*	*Flavobacteriaceae_*uncl			♦		♦	
	*Sphingobacteriia*	*Chitinophagaceae*	*Flavisolibacter*			♦		♦	
*Firmicutes*	*Bacilli*	*Bacillaceae_*1	*Bacillaceae_*1_uncl			♦		♦	
		*Bacillales_*uncl	*Bacillales_*uncl			**♦♦**		♦	
		*Carnobacteriaceae*	*Dolosigranulum*	♦			♦		
		*Carnobacteriaceae*	*Granulicatella*			♦		♦	
	*Clostridia*	*Clostridiales_*Incertae_Sedis_XI	*Anaerococcus*		♦	**♦♦**			
		*Lachnospiraceae*	*Dorea*			♦		♦	
		*Lachnospiraceae*	*Lachnospiraceae_*uncl	**♦♦**		**♦♦♦**		♦	
		*Ruminococcaceae*	*Faecalibacterium*	♦		**♦♦♦♦**			
		*Ruminococcaceae*	*Ruminococcaceae_*uncl			**♦♦**			
		*Peptostreptococcaceae*	*Clostridium_*XI			♦		♦	
	Firmicutes_uncl	Firmicutes_uncl	Firmicutes_uncl			♦		♦	
Proteobacteria	Alphaproteobacteria	Alphaproteobacteria_uncl	Alphaproteobacteria_uncl			♦		♦	
		Caulobacteraceae	*Brevundimonas*	♦		♦			
		Rhizobiales_uncl	Rhizobiales_uncl	♦		♦			
		Rhizobiaceae	*Rhizobium*			♦		♦	
	Betaproteobacteria	Comamonadaceae	Comamonadaceae_uncl			**♦** **♦♦**		♦	
		Sutterellaceae	*Sutterella*		♦	♦			
		uncl_Betaproteobacteria	uncl_Betaproteobacteria		♦	♦			
	Epsilonproteobacteria	Moraxellaceae	*Acinetobacter*			♦	♦	♦	
		Enterobacteriaceae	Enterobacteriaceae_uncl			**♦♦**		♦	
		Gammaproteobacteria_uncl	Gammaproteobacteria_uncl			**♦♦**		♦	

Specific microbial genera were associated with olfactory function subcategories: discrimination, threshold and identification. Only those genera that appeared at least at two conditions, or their correlation was found based on several OTUs are shown. Number of diamonds in the columns reflect number of associated OTUs (table with full information of associated OTUs is given in the Supplementary Table 3). Normosmic score was defined as ≥6.25 for threshold, ≥10 for discrimination, and ≥11 for identification (see methods section). Microbial taxa associated with with olfactory function subcategories are defined using LEfSe (Linear discriminant analysis Effect Size^43^) algorithm.

A number of signatures from bacterial taxa were associated with hyposmic threshold, low discrimination and low identification performance; these included mainly certain *Actinobacteria*, *Bacteroidia*, *Bacilli*, *Clostridia* and *Proteobacteria*. In particular signatures of *Corynebacterium* and *Faecalibacterium* appeared frequently as a biomarker for reduced odor discrimination and threshold, while *Comamonadaceae* and *Enterobacteriaceae* were significantly more abundant in nasal microbiome of study subjects with reduced odor threshold and identification. Signatures of genera associated with low performance in all three olfactory subcategories (T, D, I) belonged to *Porphyromonas* and unclassified *Lachnospiraceae* (Table 2 and Supplementary Table 3)^44^.

## Discussion

In this study, we explored the relationship between olfactory function and nasal microbiome in 67 healthy volunteers. The aim was to determine whether: (i) olfactory epithelium microbiome community composition and diversity differ between normosmic and hyposmic individuals, (ii) specific bacteria therein are associated with above average or impaired olfactory function, and (iii) other factors correlate with a specific microbiome composition. Our results show that olfactory function was potentially connected to nasal microbiome community composition, as the microbiomes of normosmic and hyposmic study subjects differed significantly in RDA analyses and regarding their alpha diversity. Moreover, we could also detect nearly significant differences in the microbiome composition of subjects with normal, and those with particularly good olfactory function, based on odor discrimination scores. 

Subjects with lower olfactory threshold scores had a more diverse nasal microbiome compared to those with normal odor thresholds. In general, a more diverse microbial community is accompanied by a wider range of microbial functions; this is usually acknowledged as a positive feature. Butyrate-producing *Faecalibacterium* or *Porphyromonas* in particular were strongly associated with reduced olfactory function in our study. *Porphyromonas* species are well known as butyrate-producing components of the human oral microbiome, whereas *Corynebacterium* representatives, which were correlated with reduced odor discrimination and threshold, are abundant on the skin, but, like Comamonadaceae, are also widely distributed in other human niches^45^. Futhermore, Lachnospiraceae, *Faecalibacterium* and Enterobacteriaceae, all associated with hyposmia, are typical gut microorganisms, also capable of butyric acid formation^45^.

Butyric acid, a typical product of anaerobic fermentation in the colon, plays a major role in energy balance and is involved in numerous body processes^46–48^. Characterized by a very strong and unpleasant odor, it can be detected in concentrations as low as 10 parts per million^49^. Commonly used as an odorant in chemosensory testing it has been investigated extensively as a stimulus. It has been reported that certain dietary restrictions can lead to butyric acid avoidance in mice^50^.

The microbiome has been shown to modulate the physiology of the olfactory epithelium, potentially changing the response to odorant stimulation^30^. Furthermore, olfactory receptors have been reported to detect microbial signals such as short-chain fatty acids^51^ hypothetically leading to changed olfactory perception, and it seems possible that by producing strong-smelling compounds such as butyric acid, microorganisms within the nasal microbiome could impact on olfactory perception and thus also on appetite. The sense of smell plays a major role in eating behavior, including anticipation and stimulation of appetite^52^, as well as flavor perception^8,53,54^. In our study, reflecting a healthy population we did not see a correlation of BMI with olfactory function, but a tendency towards a differing nasal microbiome between those with normal, high and low BMI. However, this relationship of BMI, body weight and olfactory function is also reflected in the literature: On one hand, individuals with an altered olfactory sense report alterations of dietary behavior and their BMI and olfactory function are negatively correlated^15,55^. On the other hand nutritional states are known to affect odor detection abilities due to hormonal and enzyme-related appetite regulation, and for patients with extreme weight/eating disorders, there is a negative impact on olfactory function for extremely high BMI and a positive impact for extremely low BMI (see for example^56–63^). Olfactory performance measures are known to be influenced by various molecules that modulate food intake^64^. The appetite-stimulating molecule ghrelin has been hypothesized to act as a moderator in emotional eating behavior, thereby influencing BMI^60^. Our finding that microbiome distribution differed with BMI level, although not quite statistically significant, might therefore be a direct result of this interaction.

Our study provides first insights into a potential correlation between olfactory function and olfactory epithelium microbiome composition. Future investigations will need to clarify whether the higher abundance of butyric acid-producing microorganisms in hyposmic individuals are a result or a cause of reduced olfactory function. Manipulation of microbiome content by using probiotics or fecal transplants has been shown to be beneficial for treating several gastrointestinal diseases associated with gut dysbiosis^65,66^. Whether altering the nasal microbiome in diseases in which the microbiota of the nasal cavity is affected, such as chronic rhinosinusitis, is feasible or has any therapeutic potential remains to be investigated^49^.

## Methods

### Subjects

Sixty-seven volunteers were recruited by means of public bulletins and announcements from the University of Graz and the Medical University of Graz, as well as announcements in local and upper regional newspapers. The following exclusion criteria were applied: consumption of antibiotics/probiotics within the last month before testing, acute hay fever, pollen allergies or common cold at the time of testing, nasal polyps, chronic neurologic, psychiatric or other severe disease, use of nasal spray on the day of testing, and age outside the specified range of 18–45 years. The study was evaluated and approved by the local ethics committee of the University of Graz (reference number GZ. 39/80/63 ex 2014/15) according to the Declaration of Helsinki. All study subjects provided an informed consent before participation.

### Olfactory function testing

For all subjects, olfactory function was assessed using the Sniffin’ Sticks battery (Burghart Instruments, Wedel, Germany) following the standardized TDI (threshold, discrimination, identification) procedure^67^, see following section for details. An ENT physician examined the nasal cavity of each participant and obtained nasal swabs (Ultra minitip flocked swab, Copan, USA) from the olfactory mucosa, located at the ceiling of the nasal cavity (Supplementary Figure 7.). Samples were stored immediately on dry ice and transferred to Medical University of Graz for further analyses. The sequence of olfactory measurements and ENT examination was counterbalanced across participants.

The Sniffin’ Sticks battery consists of pen-like devices filled with odorants^67–69^. Three subtests designed to assess orthonasal olfactory function were carried out: detection threshold (the lowest concentration at which an odor can be perceived), odor discrimination (ability to distinguish between odors) and odor identification (ability to assign names to odors). The odor detection threshold was identified for each participant using a three-alternative, single-staircase, forced-choice procedure with the odorant n-butanol. Thus, participants were presented with triplets of odorant pens and asked to identify the pen containing n-butanol amongst two blank distractor pens. In the second subtest, odor discrimination ability was assessed using 16 triplets of odorants: within each triplet, two pens contained the same, while the third pen contained a different, odorant. In a forced-choice procedure, the participants were asked to detect the odd pen for each triplet. During the odor identification task, participants were presented with 16 common odors. Using a multiple choice answering format, they were asked to select which of four odor labels matched each presented odor.

Possible scores range from 1–16 for the detection threshold subtest and 0–16 for the other two subtests. The total TDI score is obtained by summing the scores for all three subtests. Normosmia is defined by a TDI score of ≥31, hyposmia by a TDI score 17 to 30.75 and anosmia a TDI score of <17^70^. The normosmic group was subdivided into average normosmics and good normosmics, using a cutoff TDI score ≥35.5 (median split). We also evaluated the association between olfactory function and microbiome separately for odor threshold, discrimination, and identification scores. The cutoff used to differentiate normosmics from hyposmics was ≥6.25 for threshold, ≥10 for discrimination, and ≥11 for identification^70^. Average normosmics and good normosmics were differentiated using a dividing value of ≥8.9 for threshold, ≥13 for discrimination, and ≥14 for identification (score higher than 50% of the normosmic group).

### Statistical analysis

To evaluate whether olfactory scores were related to age or BMI, we calculated non-parametric Spearman’s rho correlations between these measures, as some parameters were not normally distributed. We also assessed whether olfactory scores, age or BMI differed between male and female subjects using two-sample t-tests. Statistical analyses were performed using the Statistical Package for the Social Sciences, Version 24.0 (SPSS, Chicago, Illinois). An alpha value of 0.05 was considered as statistically significant.

### Microbiome analysis

The sample tubes were placed on dry ice immediately after sampling, then samples were stored at −80 °C until further processing.

Genomic DNA was extracted from the swabs using FastDNA SPIN Kit (MP Biomedicals, USA) according to manufacturer’s instructions. Concentration of the isolated DNA was quantified with Qubit dsDNA HS Assay Kit (Thermo Fisher Scientific, USA). Variable region V4 of 16S rRNA gene was amplified with universal PCR primers 515F (5′-GTGCCAGCMGCCGCGGTAA-3′) and 806 R (5′-GGACTACHVGGGTWTCTAAT-3′)^71^ using TaKaRa Ex Taq polymerase (Clontech, Japan) and 10–20 ng template in each reaction. Cycling conditions consisted of an initial denaturation at 94 °C for 3 min, followed by 35 cycles of denaturation (94 °C 45 sec), annealing (50 °C 60 sec), extension (72 °C 90 sec), and a final extension at 72 °C for 10 min. The resulting fragments were visualized in 3% agarose gel, and sequenced at ZMF Core Facility Molecular Biology in Graz, Austria, using the MiSeq platform.

### Microbiome data processing

To analyze the microbial community structure and taxonomic diversity, the obtained raw reads were processed using mothur version 1.36.1^72^ following the Standard Operation Procedure (SOP). In short, the paired end reads were joined together, the produced sequences were quality checked (minimum length 200, maximum length 300, maximum number of homopolymers 8) and aligned against the SILVA 123 database^73^. Good-quality sequences were then pre-clustered and chimeric sequences removed. Taxonomic assignment was carried out by querying the sequence reads against a trainset14_032015 reference database, after which the sequences were clustered into OTUs (threshold 0.03 dissimilarity) using average neighbor algorithm. A biom table was constructed for downstream analyses. OTUs represented by ≤5 sequences were removed from the analysis.

To evaluate alpha and beta diversities and differences in community composition, and visualize the results, we applied Calypso (Version 5.8), an online platform for mining, visualizing and comparing multiple microbial community composition data (cgenome.net/calypso). Total-sum normalization was applied for 16 S rRNA gene data. RDA and PCA plots were created with Calypso Multivariate tool, and inverse simpson indices were calculated and visualized with Calypso Diversity tool. The differences in alpha diversity were analyzed using anova. Individual OTUs were correlated with total TDI scores using regression analysis (Pearson correlation).

## Declarations

### Ethics approval and concent to participate

The study was evaluated and approved by the local ethics committee of the University of Graz (reference number GZ. 39/80/63 ex 2014/15) according to the Declaration of Helsinki. All study subjects provided an informed consent before participation.

### Availability of data and materials

The dataset supporting the conclusions of this article is available in The European Nucleotide Archive (ENA) under study accession number PRJEB20526.

## Electronic supplementary material

Additional file 1

Additional file 2

Additional file 3
