# Supplementary material for: The nasal microbiome mirrors and potentially shapes olfactory function
Source: Sci Rep. 2018 Jan 22;8:1296. doi: 10.1038/s41598-018-19438-3 (PMC5778015; doi:10.1038/s41598-018-19438-3)
Supplement: Supplementary file 1 — Additional file 1 [file 41598_2018_19438_MOESM1_ESM.pdf]

The nasal microbiome mirrors and potentially shapes olfactory function

Kaisa Koskinen[1,2], Johanna L. Reichert[2,3], Stefan Hoier[4], Jochen Schachenreiter[5], Stefanie Duller[1],

Christine Moissl-Eichinger[1,2,*], Veronika Schöpf[2,3,*]

Supplementary results

Supplementary figures

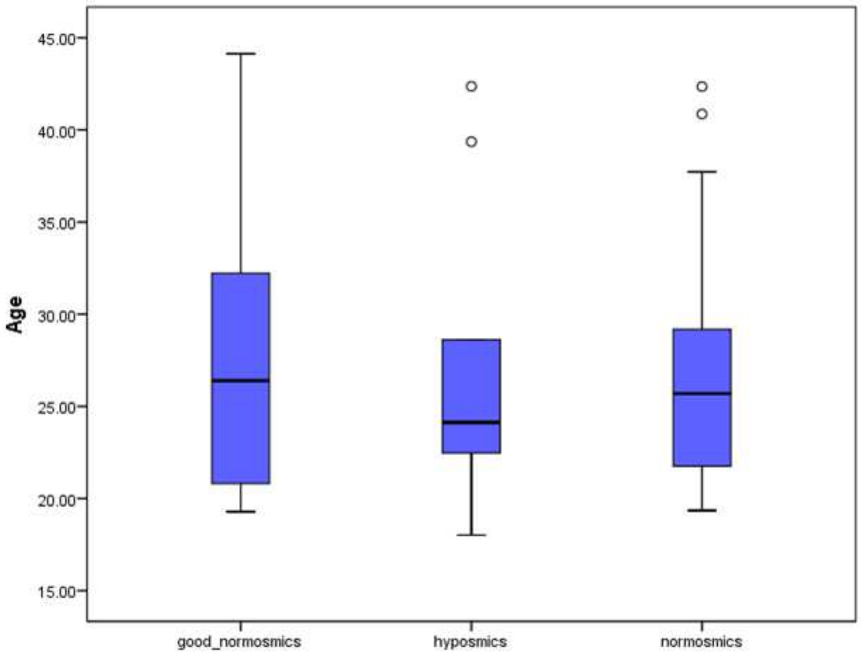

Supplementary Figure 1. Boxplots showing the distribution of age in each of the three groups (good normosmics, hyposmics and normosmics based on total TDI) Outliers are marked with circles.

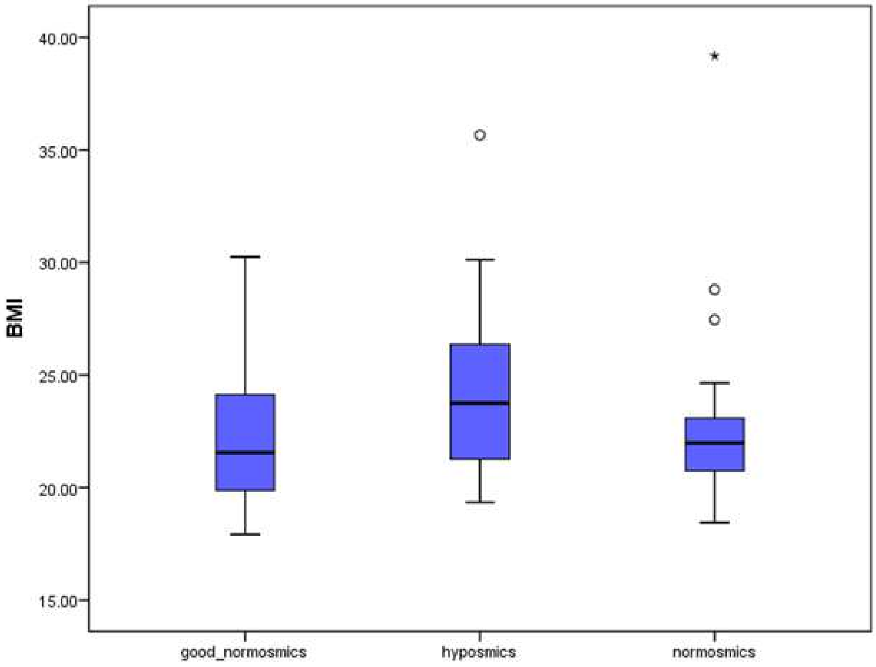

Supplementary Figure 2. Boxplots showing the distribution of BMI in each of the three groups (good normosmics, hyposmics and normosmics based on total TDI). Outliers are marked with circles, far outliers with stars.

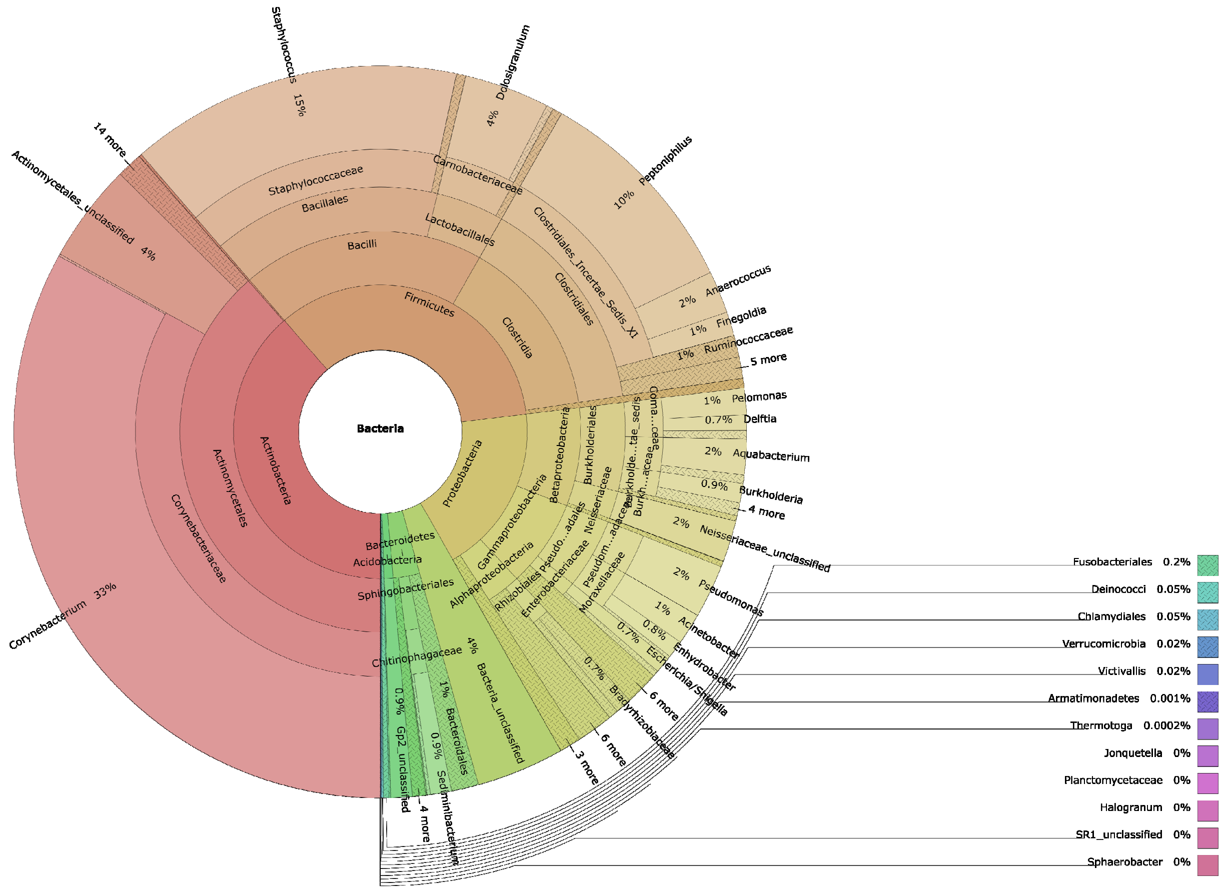

Supplementary Figure 3. Hierarchical display (Krona diagram) of the human nasal bacteriome as revealed in this study, from all hyposmic participants.

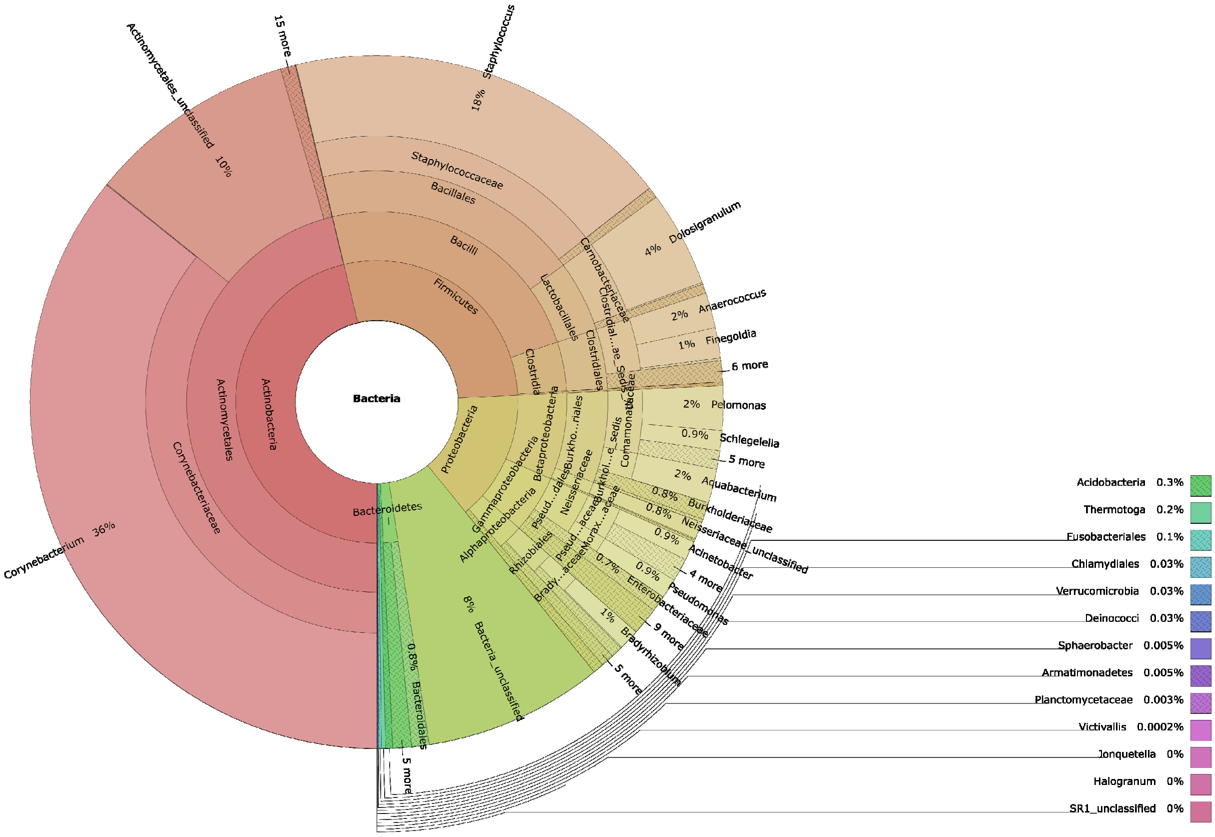

Supplementary Figure 4. Hierarchical display (Krona diagram) of the human nasal bacteriome as revealed in this study, from all average normosmic participants.

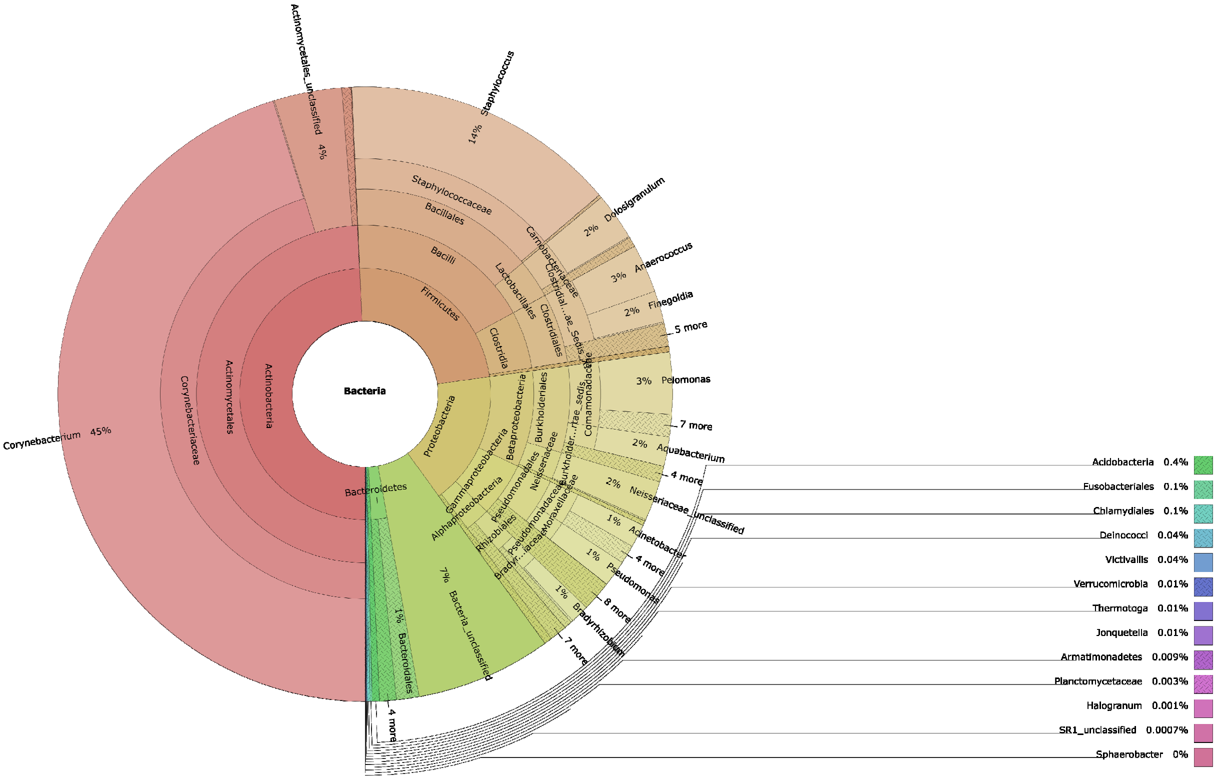

Supplementary Figure 5. Hierarchical display (Krona diagram) of the human nasal bacteriome as revealed in this study, from all good normosmic participants.

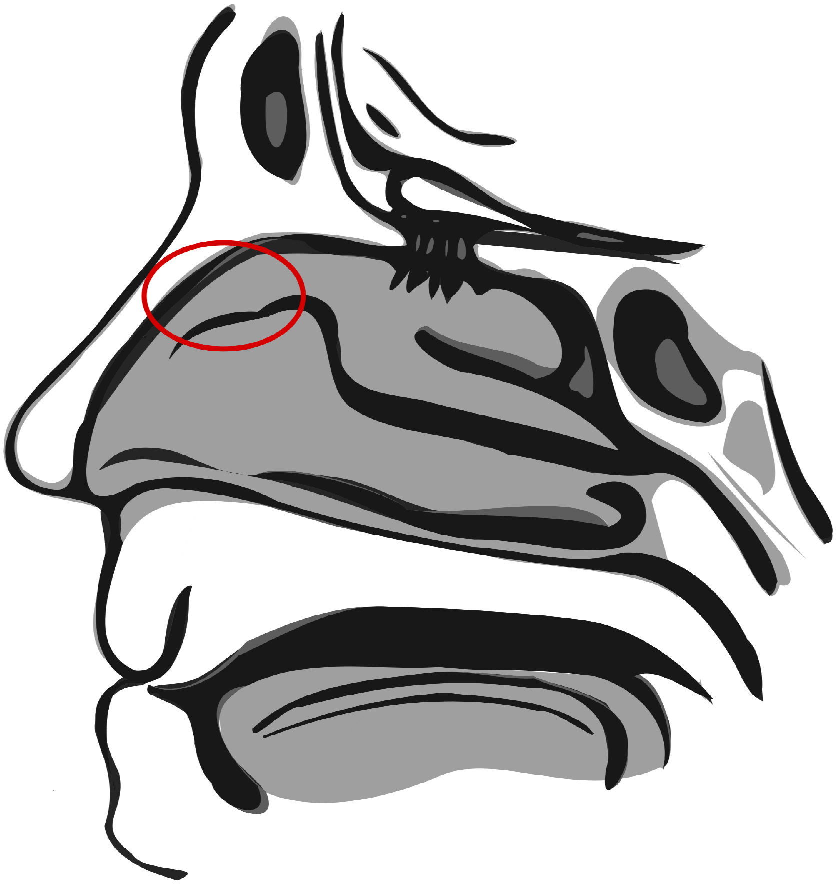

Supplementary Figure 6. Schematic picture of the nasal cavity. Nasal microbiome samples were taken from the olfactory mucosa in the nasal cavity.

Supplementary tables

Supplementary Table 1. Olfactory performance scores, bodyweight and sex of study subjects

	N	% female	*mean* TDI (*SD*)	*mean* age (*SD*)	*mean* weight (*SD*)	*mean* BMI (*SD*)	%normal weight	%over-weight	%under-weight
TDI									
Hyposmics	10	50.0	26.5 (3.9)	27.1 (7.9)	75.3 (15.1)	24.9 (5.0)	60.0	40.0	0.0
Average normosmics	28	85.7	33.4 (1.3)	26.9 (6.1)	65.6 (14.1)	22.7 (3.9)	85.7	10.7	3.6
Good normosmics	29	72.4	37.6 (2.1)	27.9 (7.4)	64.8 (11.3)	22.0 (2.9)	79.3	10.3	10.3
Threshold									
Hyposmics	5	60.0	26.7 (6.0)	26.7 (9.2)	75.6 (24.4)	25.2 (7.4)	40.0	40.0	20.0
Ave normosmics	25	72.0	33.1 (3.3)	27.1 (6.6)	66.4 (10.6)	22.9 (2.7)	76.0	20.0	4.0
Good normosmics	37	78.4	36.0 (3.4)	27.5 (6.9)	65.7 (13.3)	22.2 (3.8)	86.5	8.1	5.4
Discrimination									
Hyposmics	14	71.4	30.1 (3.7)	26.0 (6.1)	65.7 (7.7)	21.9 (2.4)	78.6	14.3	7.1
Ave normosmics	12	91.7	32.4 (5.0)	28.4 (7.8)	65.9 (19.7)	24.0 (6.1)	66.7	25.0	8.3
Good normosmics	41	70.7	36.2 (2.9)	27.5 (6.9)	67.3 (13.0)	22.6 (3.3)	82.9	12.2	4.9
Identification									
Hyposmics	8	62.5	27.6 (5.0)	28.7 (8.6)	76.8 (15.9)	25.5 (5.6)	62.5	37.5	0.0
Ave normosmics	32	81.3	33.8 (3.2)	26.2 (6.2)	65.0 (9.7)	22.2 (2.4)	81.3	12.5	6.3
Good normosmics	27	70.4	36.6 (3.0)	28.2 (7.1)	65.8 (15.5)	22.5 (4.3)	81.5	11.1	7.4
